# Toxic Metals, Non-Metals and Metalloids in Bottom Sediments as a Geoecological Indicator of a Water Body’s Suitability for Recreational Use

**DOI:** 10.3390/ijerph20054334

**Published:** 2023-02-28

**Authors:** Martyna A. Rzetala, Robert Machowski, Maksymilian Solarski, Daniel Bakota, Arkadiusz Płomiński, Mariusz Rzetala

**Affiliations:** 1 Institute of Earth Sciences, Faculty of Natural Sciences, University of Silesia in Katowice, Będzińska 60, 41-200 Sosnowiec, Poland; 2 Institute of Social and Economic Geography and Spatial Management, Faculty of Natural Sciences, University of Silesia in Katowice, Będzińska 60, 41-200 Sosnowiec, Poland; 3 Faculty of Social Sciences, Jan Długosz University in Częstochowa, Waszyngtona 4/8, 42-200 Częstochowa, Poland

**Keywords:** bottom sediments, trace elements, toxic metals, heavy metals, water bodies, recreation, Silesian Upland

## Abstract

The study of bottom sediments was conducted within the basins of water bodies used for recreational purposes (e.g., bathing, fishing and diving) in the Silesian Upland and its periphery in southern Poland. Various concentrations of trace elements were found in bottom sediments, reflected by the following levels: Pb (30–3020 mg/kg), Zn (142–35,300 mg/kg), Cd (0.7–286 mg/kg), Ni (10–115 mg/kg), Cu (11–298 mg/kg), Co (3–40 mg/kg), Cr (22–203 mg/kg), As (8–178 mg/kg), Ba (263–19,300 mg/kg), Sb (0.9–52.5 mg/kg), Br (1–31 mg/kg), Sr (63–510 mg/kg) and S (0.001–4.590%). These trace elements are present in amounts that usually exceed those found in other bodies of water or are sometimes even unprecedented among bodies of water in the world (e.g., cadmium—286 mg/kg, zinc—35,300 mg/kg, lead—3020 mg/kg, arsenic—178 mg/kg). It was found that bottom sediments were contaminated to varying degrees with toxic metals, metalloids and non-metals, as evidenced by the values of geoecological indicators, i.e., the geoaccumulation index (−6.31 < *I_geo_* < 10.90), the sediment contamination factor (0.0 ≤ $C_{f}^{i}$ < 286.0), the sediment contamination degree (4.6 < *C_d_* < 513.1) and the ratios of the concentrations found to the regional geochemical background (0.5 < *I_RE_* < 196.9). It was concluded that the presence of toxic elements (e.g., lead, zinc, cadmium, chromium, strontium and arsenic) in bottom sediments should be taken into account when classifying water bodies as suitable for recreational use. A maximum ratio of the concentrations found to the regional geochemical background of *I_RE_* ≤ 5.0 was proposed as the threshold for the permissibility of recreational use of water bodies. The water bodies used for recreational purposes in the Silesian Upland and its periphery do not meet the geoecological conditions for safe use in terms of recreation and leisure activities. Forms of their recreational use that directly affect the participants’ health (e.g., fishing and the consumption of fish and other aquatic organisms) should be abandoned.

## 1. Introduction

Metals (lead—Pb, zinc—Zn, cadmium—Cd, nickel—Ni, copper—Cu, cobalt—Co, chromium—Cr, barium—Ba, strontium—Sr), metalloids (antimony—Sb, arsenic—As) and non-metals (bromine—Br, sulfur—S) are commonly found in the environment, albeit unevenly distributed in the Earth’s crust. They are present in varying amounts, from trace concentrations to levels corresponding to those of major elements. Their presence in the environment are consequences of natural processes (e.g., geological ones), but often also the results of human activity and of economic processes related to the development of agriculture, urbanization, industry and services [1,2]. Human activity, which has intensified particularly since the industrial revolution [3,4], has played an increasingly important role in shaping the conditions for the natural circulation of elements such as Cu, Pb, Zn, Ni, Cd, Co, Cr, Ba, As, Sb, Br and S. It is widely believed that trace elements are essential for the proper functioning of the human body [5]. However, in higher concentrations, they are toxic to organisms, including humans [6]. Some metals (e.g., zinc, copper and chromium), metalloids and non-metals are considered necessary for organisms to thrive as trace elements; and others (e.g., lead and cadmium) are considered entirely unnecessary and even harmful and toxic to plants, animals and humans, especially in high concentrations [1]. Such metals as lead [7,8] and cadmium [9,10] especially, which are harmful in any amounts, exhibit a high degree of toxicity, interfering with the normal course of life processes in the human body [11].

For the aforementioned reasons, the presence of metals, metalloids and non-metals in the environment is carefully monitored. Since they provide a good indicator of the characteristics of the surrounding environment, bottom sediments of lakes and other water bodies are often subject to geochemical studies [12,13]. Metals, metalloids and non-metals accumulate in bottom sediments in amounts commensurate with their content in catchments (area from where water is collected) and in the atmosphere. Therefore, they migrate into the bottom sediments from various sources, i.e., together with surface and underground flows, from the coastal zone, from atmospheric deposition and also as a result of sedimentation of autochthonous plankton and vegetation. Their concentrations should correspond to levels considered natural, i.e., to geochemical background levels. A decisive role in shaping the geochemical background is played by the geological formations present within the catchment area. Where human pressure is present, concentrations of metals, metalloids and non-metals can significantly exceed geochemical background levels. Comparing concentrations of the elements tested to the geochemical background makes it possible to assess levels of metals, metalloids and non-metals as indicators not only of the characteristics of the surrounding environment, but also of the suitability of water bodies, e.g., as a source of water supply for irrigation in agriculture, for use in production processes, for human consumption and for recreation and leisure purposes.

In the modern world, water bodies are used in many ways. Among their uses are active recreation and leisure [14,15,16]. The types and forms of recreational activities carried out within water bodies are determined by the quality of water (pollution level) and its impact on health [17,18]. Pure water, which translates to the abundant presence of many species of fish, promotes the development of recreational fishing and sometimes even fishing tourism [19,20,21,22]. Water bodies that are free of pollution are more attractive, which in turn encourages the construction of specialized recreational infrastructure that makes full use of the potential of these water bodies and their surroundings for leisure purposes [23,24,25,26,27,28,29]. Apart from their positive aspects, recreational activities may also adversely affect water bodies. In particular, this includes all forms of recreation where boats, jet skis and yachts equipped with internal combustion engines are used. Oil spills, sewage, noise and the use of anti-fouling paints not only pollute bodies of water, but also threaten wildlife. Some water bodies used for recreational purposes have pollution from urban and industrial centers within the proper ranges—e.g., air pollution and domestic, industrial and municipal sewage discharges [30,31]. A number of research questions arise in relation. Do bottom sediments of recreationally used water bodies vary in terms of their metal and non-metal concentrations? Are these sediments contaminated? Can the presence of metals and non-metals in bottom sediments be considered a geoecological indicator of the suitability of water bodies for recreational use?

The aim of the study was to assess the feasibility of using the presence of metals, metalloids and non-metals in bottom sediments as a geoecological indicator of the suitability of inland water bodies for recreational use, and thus to evaluate the contents of these trace elements as indicators of anthropogenic pollution. The studies conducted are important because water bodies are usually classified as suitable for recreational purposes solely on the basis of water quality assessments, without considering the geoecological condition of their bottom sediments. It is precisely the quality of bottom and coastal sediments of water bodies used for recreational purposes in the Silesian Upland that is responsible for the occurrence of local ecological disasters, e.g., the elimination of plant and animal species [12,32], mass mortality of fish and other aquatic organisms [12], eutrophication and even hypertrophication [33]. Thus, the proposed research is not just of cognitive importance—related to the explaining the origins and effects of the presence of toxic metals in the sediments of water bodies used for recreational purposes—but it also has applications, making it possible to assess whether the body of water in question is suitable for recreation. Among the many regional reports from the Silesian Upland and its periphery, and also from worldwide research on the role of metals, non-metals and metalloids in bottom sediments, the research presented in this paper represents a completely new proposal for assessing the suitability of water bodies for recreational purposes on the basis of geoecological indicators pertaining to sediments. In this respect, this work appears to fill important cognitive, methodological and application gaps.

## 2. Study Area

**Figure 1:**
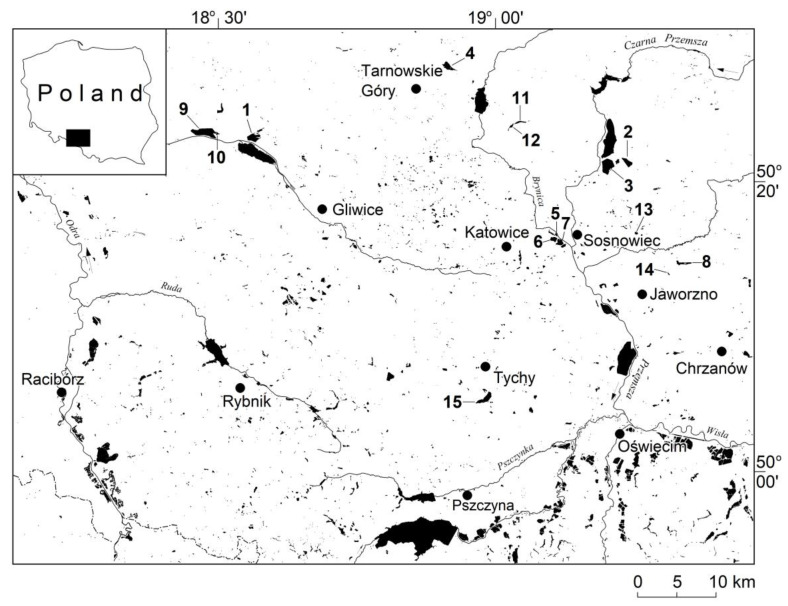
Locations of the water bodies used for recreational purposes covered by the study in the Silesian Upland and its periphery: 1—Dzierżno Małe, 2—Pogoria I, 3—Pogoria III, 4—Chechło, 5—Stawiki, 6—Morawa, 7—Gliniak, 8—Sosina, 9—Pławniowice, 10—Mały Zalew, 11—Rogoźnik II, 12—Rogoźnik I, 13—Balaton, 14—Koparki, 15—Paprocany.

Water bodies are sedimentation basins with known morphometric parameters and ages, which are filled with bottom sediments. The study of bottom sediments was conducted within the basins of 15 water bodies used for recreational purposes (e.g., bathing, fishing, water sports, diving and canoeing). These are situated in the Silesian Upland and its periphery in southern Poland (Figure 1, Table 1).

The Silesian Upland, together with its periphery, is coextensive with the so-called Upper Silesian Anthropogenic Lake District, where there are about 4700 water bodies with a total area of 185.4 square kilometers [34]. At the same time, it is a densely populated area, which is related to the region’s industrial development and urbanization that dates back more than two centuries. The Silesian Upland is among the regions of Europe with the greatest abundance of energy resources and metal ores. The Upper Silesian Industrial Region (Górnośląski Okręg Przemysłowy—GOP), which developed there, was one of the continent’s largest industrial districts (and at the same time, the largest environmental disaster areas) for decades. This industrial legacy, extensive wooded areas and numerous bodies of water form the basis of the industrial tourism that is developing in the region today, and providing settings for various forms of ecotourism and recreation for the conurbation’s approximately 2.2 million residents [14]. For the aforementioned reasons, the water bodies used for recreational purposes in the Silesian Upland and its periphery function under varying degrees of human (e.g., industrial and agricultural) pressure, which is reflected in the varied forms of their catchment development and use.

## 3. Materials and Methods

A total of 43 samples were collected from the bottom sediments of the water bodies selected for the study, taking into account the morphometric differences existing between the water bodies (i.e., the shapes of their basins, dimensions and depth variation) and the principle of uniform sampling of the sediment cover. Samples were collected in the deepest parts of the water bodies and in zones corresponding to their average depth, and additionally in bays, if these were present within the water bodies. These are the locations recognized in limnological studies as the most representative for reconstructing the occurrence and quantitative and qualitative variation of bottom sediments, designated for sampling based on bathymetric charts (maps showing depth distribution in lakes). Bottom sediment samples were collected using the Beeker sediment core sampler (04.20.S.A. version, manufactured by Eijkelkamp). Samples were additionally collected using van Veen samplers with a capacity of 1.25 dm^3^ or 2.50 dm^3^. Scoops were used where low sediment thickness prevented sampling with a core sampler (for instance, in the Koparki water body, which is less than 20 years old—sediment thickness is negligible, making it impossible to effectively use a pneumatic core sampler). Using the material sampled from a given vertical profile, a mixed sample representative of the site in question was prepared. The thickness of sediments in the anthropogenic water bodies studied is low (among other things, due to their young age), and these bottom sediments have the characteristics of sapropel that has been mixed both due to natural factors and through human activity. The mixed samples were subsequently placed in polyethylene bags and transported to the laboratory.

At the laboratory of the Institute of Earth Sciences at the University of Silesia in Sosnowiec (Poland), bottom sediment samples were dried at 105 °C until a constant mass was obtained, and then they were homogenized using a mortar and pestle. After the material had been ground, the <0.063 mm fraction was isolated using chemically inert sieves, and was subsequently subjected to geochemical analysis. This material came from the homogenized total mass of the sample. It was decided to choose fine sediment for the study due to the fact that it absorbs toxic metals, non-metals and metalloids to the greatest extent (in contrast to the negligible role of the coarse sediment in their accumulation). The samples prepared in this manner were subsequently subject to analyses at Activation Laboratories Ltd. at Ancaster (Canada). Chemical composition was determined using inductively coupled plasma atomic emission spectrometry (ICP-OES) and instrumental neutron activation analysis (INAA) in accordance with the standards applied at Activation Laboratories Ltd. [12]. Mercury content was determined using the Cold Vapour FIMS (Perkins Elmer FIMS 100) method. For the purpose of calculating one of the indicators, polychlorinated biphenyls (PCB) levels were estimated on the basis of the results of water sediment monitoring in the region (conducted by the State Inspectorate of Environmental Protection).

The concentrations of Cu, Pb, Zn, Ni, Cd and S were determined using the ICP-OES method following the complete dissolution of 0.25 g aliquots. Each sample aliquot was digested using a mixture of HClO_4_, HNO_3_, HCl and HF at 200 °C until fuming, and subsequently diluted with aqua regia [12,35]. During ICP analysis, reagent blanks with and without the lithium borate flux were analyzed alongside the method reagent blank. Interference correction verification standards were subject to analysis as well [12,35]. For calibration purposes, USGS and CANMET certified reference materials (two standards for each group of ten samples) were used in order to bracket sample groups. Moreover, internal standards were added to the sample solution, which was then subject to further dilution. When introducing the sample into the Perkin Elmer SCIEX ELAN 6000 mass spectrometer, a proprietary methodology was used [35]. Ba and Sr content were determined using the same method. Samples were prepared and analyzed in batches; each batch contained a method reagent blank, certified reference material and 17% replicates. For analysis purposes, the samples were mixed with lithium metaborate and lithium tetraborate, and then fused in an induction furnace. The resulting molten material was poured into a solution of 5% nitric acid containing an internal standard, and then mixed continuously for approximately 30 min until completely dissolved. The samples were subsequently tested for selected trace elements using a combination simultaneous/sequential Thermo Jarrell-Ash ENVIRO II ICP spectrometer [12,35]. The analyses conducted exhibited the following precision and accuracy levels: (a) at the lower detection limit: ±100%; (b) at 10 times the lower detection limit: ±15–25%; (c) at 100 times the lower detection limit: better than 10% [12,35].

The INAA method was used to determine the presence of As, Br, Co, Cr and Sb. One-gram aliquots were each placed in a polyethylene vial and irradiated with flux wires and an internal standard (one per 11 samples) at a thermal neutron flux of 7 × 1012 *n* cm^−2^ s^−1^. After seven days had passed (to allow Na-24 to decay), the samples were counted using a high-purity Ge detector offering a resolution higher than 1.7 KeV for the 1332 KeV Co-60 photopeak [35]. The decay-corrected activities were compared to a calibration obtained using multiple certified international reference materials, with the use of flux wires. Between 10% and 30% of the samples were rechecked by repeating the measurement. The standard was only used to check measurement accuracy and not for calibration purposes [35]. The analyses conducted exhibited the following precision and accuracy levels: (a) at the lower detection limit: ±100%; (b) at 10 times the lower detection limit: ±10–15%; (c) at 100 times the lower detection limit: better than 5% [12,13,14,15,16,17,18,19,20,21,22,23,24,25,26,27,28,29,30,31,32,33,34,35].

The lower quantification limit varied and was as follows: 0.2 mg/kg for Sb, 0.5 mg/kg for Cd, 1.0 mg/kg for Cu, Zn, Ni, Co, Cr and Br, 2.0 mg/kg for Sr and As, 3.0 mg/kg for Ba, 5.0 mg/kg for Pb, 0.5 μg/kg for Hg, and 0.001% for S [12,35].

To assess the geoecological significance of trace element concentrations and their quantitative variation in the bottom sediments of the water bodies used for recreation purposes, the following indicators were used: the geoaccumulation index (Equation (1)) developed by G. Müller [36,37]; the contamination degree index and contamination factor of bottom sediments (Equation (2)) developed by L. Håkanson [38]; and the ratio of concentrations to the regional geochemical background (Equation (3)) [1].

The geoaccumulation index (Equation (1)) is a popular indicator for assessing the degree of contamination of bottom sediments, which is used worldwide in geochemical studies. There are several classes of sediment quality: class 0—practically uncontaminated (*I_geo_* ≤ 0.0); class I—uncontaminated to moderately contaminated (0.0 < *I_geo_* ≤ 1.0); class II—moderately contaminated (1.0 < *I_geo_* ≤ 2.0); class III—moderately to heavily contaminated (2.0 < *I_geo_* ≤ 3.0); class IV—heavily contaminated (3.0 < *I_geo_* ≤ 4.0); class V—heavily to extremely contaminated (4.0 < *I_geo_* ≤ 5.0); and class VI—extremely contaminated (*I_geo_* > 5.0) [39,40].

(1) $$I_{geo}=\log_{2}\frac{C_{n}}{1.5\;B_{n}}\;$$

where *I_geo_*—geoaccumulation index; *C_n_*—the concentration of the element in question in bottom sediments; *B_n_*—geochemical background for the element in question; 1.5—coefficient expressing natural variation in the content of the element in question in the environment.

The contamination degree index (*C_d_*) and the contamination factor ($C_{f}^{i}$) were introduced by L. Håkanson [38] and are in fact covered by a single formula (Equation (2)). When determining the sediment contamination degree (*C_d_*), it is simply necessary to first calculate the value of the contamination factor for individual substances ($C_{f}^{i}$) [39].

(2) $$C_{d}=\overset{8}{\underset{i=1}{\sum }}C_{f}^{i}=\overset{8}{\underset{i=1}{\sum }}\frac{{\bar{C}}_{0-1}^{i}}{C_{n}^{i}}$$

where *C_d_*—the degree of contamination; $C_{f}^{i}$—the contamination factor; ${\bar{C}}_{0-1}^{i}$—the mean content of the substance in question (*i*) from superficial sediments (0–1 cm) from accumulation areas (the mixed sediment sample was considered representative of contemporary concentrations in the 0–1 cm layer due to the age of the water bodies, which was frequently no more than 40–50 years in total or since the last dredging); $C_{n}^{i}$—the standard preindustrial reference level, determined from various European and American lakes to be (in ppm): PCB = 0.01, Hg = 0.25, Cd = 1.0, As = 15.0, Cu = 50.0, Pb = 70.0, Cr = 90.0 and Zn = 175.0.

Contamination degree indicators (*C_d_*) calculated for the individual sgeubstances indicated by L. Håkanson [38]—PCBs, Hg, Cd, As, Cu, Pb, Cr and Zn—can be interpreted in accordance with the value ($C_{f}^{i}$), which indicates that contamination is absent or that it is at a low, moderate, significant or high level. The comparison is between the concentration of the substance in question in the sediment surface layer and the pre-industrial content of that substance in the sediment. The results should be interpreted as follows: $C_{f}^{i}$ < 1—no contamination or low sediment contamination; 1 ≤ $C_{f}^{i}$ < 3—moderate contamination; 3 ≤ $C_{f}^{i}$ < 6—significant contamination; $C_{f}^{i}$ > 6—very heavy contamination. In turn, the contamination degree (*C_d_*) after all $C_{f}^{i}$ indicators have been taken into account should be interpreted according to the following key proposed by L. Håkanson [38]: *C_d_* < 8—low degree of sediment contamination, 8 ≤ *C_d_* < 16—moderate degree of sediment contamination, 16 ≤ *C_d_* < 32—significant degree of sediment contamination, *C_d_* > 32—very high degree of anthropogenic sediment contamination [39]).

The ratio of element concentration to the regional geochemical background directly describes the multiple by which the natural concentration of the element in regional sediments is exceeded by the concentration of that element in the sediment sample and is described by the following formula [1,39]:

(3) $$I_{RE}=\frac{C_{BS}}{C_{RGB}}$$

where *I_RE_*—the ratio of the value measured to the regional geochemical background; *C_BS_*—the concentration of the element in question in bottom sediments; *C_RGB_*—the regional geochemical background level for the element in question in bottom sediments.

The ratio of the value measured to the regional geochemical background (*I_RE_*) calculated in this manner exceeds unity if the concentration of the element in question is higher than the regional geochemical background (the higher the concentration the higher the ratio) and is below unity when this level is not reached.

When calculating the indicators presented above, it is extremely important to refer to the value of the geochemical background, the natural content of the substance in question in the sediments. Among the many results of sediment geochemical background tests, the most recent and most commonly used values were used [41,42,43,44,45,46]. In the calculation of the geoaccumulation index (*I_geo_*), the geochemical background values for individual elements presented in Li and Schoonmaker [41] were adopted. For individual trace elements, the listed values are Cu—39.0 mg/kg, Pb—17.0 mg/kg, Zn—67.0 mg/kg, Ni—55.0 mg/kg, Cd—0.1 mg/kg, Co—17.0 mg/kg, Cr—69.0 mg/kg, Ba—570.0 mg/kg, Sr—350.0 mg/kg, As—1.0 mg/kg, Sb—0.2 mg/kg, Br—2.1 mg/kg and S—0.053%. When determining the ratio of actual element concentrations to the regional geochemical background (I_RE_), the data most representative of studies of bottom sediments of water bodies in the Silesian Upland and its periphery that were published in the regional geochemical atlas were used [42]. Geochemical background values for water sediments in the Silesian Upland for the trace elements measured were established at: Cu—15.0 mg/kg, Pb—59.0 mg/kg, Zn—259.0 mg/kg, Ni—11.0 mg/kg, Cd—2.5 mg/kg, Co—4.0 mg/kg, Cr—9.0 mg/kg, Ba—98.0 mg/kg, Sr—24.0 mg/kg, As—6.0 mg/kg and S—0.052%; no data were available for Sb and Br.

Using generalization, the main types of land use were identified in the catchment areas of the studied water bodies. Determination of the type of land use was conducted through field mapping.

## 4. Results

Trace element levels in the bottom sediments of the water bodies studied vary greatly (Table 2 and Table 3). The trace elements found exhibit considerable variation in terms of their respective levels—e.g., Cd and Sb occur in amounts ranging from tenths of a milligram per kilogram to fifty-two point five milligrams per kilogram (Sb) or two-hundred and eight six milligrams per kilogram (Cd). Pb and Zn reach concentrations ranging from several dozen (Pb) or several hundred milligrams per kilogram (Zn) to thousands of milligrams per kilogram. The elements in question are also characterized by highly variable concentrations in individual samples. Some elements (e.g., Cu, Ni, Cr, Co, As, Sr, Sb and Br) exhibited differences in their concentrations in the samples tested amounting to an order or two of magnitude, and even greater discrepancies were observed for Cd, Pb, Zn, Ba and S.

Geoaccumulation indices (*I_geo_*) calculated from the results of elemental content measurements in bottom sediment samples of recreationally used water bodies in the Silesian Upland are in the range of −6.31 < *I_geo_* < 10.90 (Table 4).

The ratio of the trace element’s measured value to the regional geochemical background reflects the concentration in bottom sediments in relation to the levels considered natural in region and also indicates the contamination level. Ratios of the values measured to the regional geochemical background (*I_RE_*) were as follows: Cu—0.7–19.9, Pb—0.5–51.2, Zn—0.5–136.3, Ni—0.9–10.5, Cd—0.3–114.4, Co—0.8–10.0, Cr—2.4–22.6, Ba—2.7–196.9, Sr—2.6–21.3, As—1.3–29.7, S—0.0–88.3 (Table 5).

**Figure 2:**
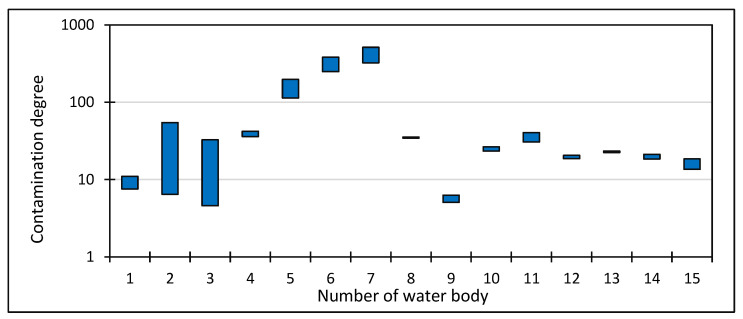
Degree of contamination of bottom sediments of water bodies used for recreational purposes in the Silesian Upland and its periphery (numbering of water bodies—see Figure 1).

The sediment contamination index ($C_{f}^{i}$) proposed by L. Håkanson [38] for the samples tested had values of 0.0 ≤ $C_{f}^{i}$ < 286.0. The second proposal of this researcher was derived from the calculated sediment contamination indices ($C_{f}^{i}$), which make up the so-called degree of sediment contamination (*C_d_*). In the case of the studied bottom sediments of water bodies used for recreational purposes in the Silesian Upland, the parameter was in the range of 4.6 ≤ *C_d_* < 513.1 (Figure 2).

**Figure 3:**
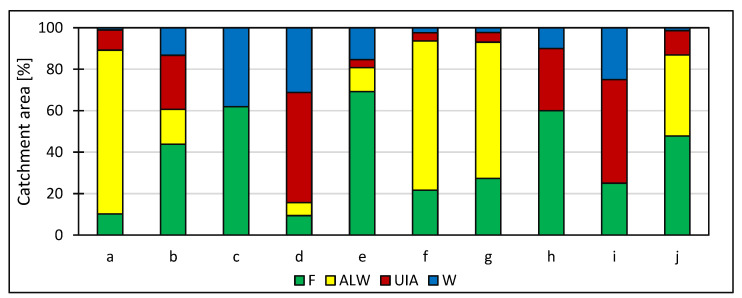
Land use forms in catchments of water bodies in the Silesian Upland and its periphery as catchment-area percentages. Land use forms: F—forest, ALW—agricultural land and wasteland, UIA—urban and industrial areas, W—water. Water body catchments (with outlets specified): a—Dzierżno Małe catchment area, b—Pogoria III catchment area (including the Pogoria I reservoir), c—Chechło catchment area 1, d—catchment areas of water bodies (including the Stawiki, Morawa and Gliniak), e—Sosina catchment area, f—Pławniowice catchment area (including the Mały Zalew reservoir), g—Rogoźnik I catchment area (including the Rogoźnik II reservoir), h—Balaton catchment area, i—Koparki catchment area, j—Paprocany catchment area.

The catchment areas of the studied water bodies are diverse in size and major forms of use (Figure 3). They were established to facilitate the recognition of the conditions for the occurrence of toxic metals, non-metals and metalloids in the bottom sediments of water bodies used for recreational purposes in the Silesian Upland and to indicate the impact of varying anthropogenic pressure in the catchment areas of these water bodies.

The Paprocany reservoir has the largest catchment area among the studied water bodies (133.1 km^2^), followed by the catchment areas of the reservoirs: Dzierżno Małe (130.6 km^2^), Pław-niowice (119.1 km^2^), Pogoria III (22.6 km^2^), Rogoźnik I (17.2 km^2^), the mouth of the Rawa River to the Brynica River (3.2 km^2^), Sosina (2.6 km^2^), Chechło (2.1 km^2^), Balaton (1.0 km^2^) and Koparki (0.4 km^2^). The largest share of urban and industrial land is present in the catchment area of the reservoirs at the mouth of the Rawa River to the Brynica River (1.7 km^2^; 53.1%)—this land is located in the vicinity of approx. 1 km from a defunct non-ferrous metal smelter. The largest share of agricultural land is in the catchment area of the Dzierżno Małe reservoir (103.2 km^2^, 79.0%). The catchment areas of the Sosina (1.8 km^2^, 69.2%) and Chechło (1.3 km^2^, 61.9%) reservoirs have the highest proportions of forests; an active zinc smelter is located some 3 km from the boundaries of the catchment area of the Chechło reservoir.

## 5. Discussion

### 5.1. Geochemical Properties of Sediments—Comparison with Literature Data

The bottom sediments of water bodies accumulate trace elements to varying degrees, and their concentrations reflect the extent of anthropogenic pollution [47,48]. This pollution can be expressed by reference to the concentration of the substances analyzed in other bodies of water in the world which are used for similar purposes, but indicators that take into account natural trace element levels are of particular importance. The analyses conducted not only revealed a number of differences between the occurrence of toxic metals, metalloids and non-metals in the bottom sediments of water bodies, but also position the region of the Silesian Upland and its periphery as unique in terms of, e.g., lead, cadmium, zinc and copper concentrations (Table 6).

Lead (Pb) is classified as a heavy metal, and owing to its properties, it has been widely used by humans since ancient times [85]. Its geochemical cycle in the environment is now determined mostly by human activity [86]. In the Silesian Upland, human use of lead dates back at least to the Middle Ages [87,88,89]. Heavy metals were found in the bottom sediments of the water bodies tested in amounts ranging from 30 to 3020 mg/kg. The highest concentrations were found in water bodies adjacent to non-ferrous smelter locations, and this can be explained by the fallout of contaminated dust from the atmosphere and the use of waste materials in the reclamation of depressions left by former mineral workings, which depressions were subsequently occupied by the Morawa, Stawiki and Gliniak water bodies. These are now used for recreational purposes, but no other water bodies in the world can match them in terms of the concentration of this toxic metal in bottom sediments [1,12].

Similar considerations as those concerning lead apply to the presence of zinc in the bottom sediments of the water bodies examined. Zinc (Zn) has been found in amounts ranging from 142 to 35,300 mg/kg, which is an amount unprecedented in the bottom sediments of not only water bodies used for recreational purposes, but also in other water bodies around the world in general—even those whose waters have been included in production cycles [12]. An example of such a water body is Lake Gusinoe in Zabaikalye Krai, which supplies water to the towns and villages in its vicinity; is used for recreation and fishing purposes; and is also a reservoir of cooling water for the neighboring power plant and receives waters from coal-mine drainage. Zinc concentrations in sediments in the central part of the lake reach 74.2 mg/kg, rise to 555 mg/kg in the vicinity of the power plant and have a maximum of 598 mg/kg in the outflow zone [53].

Cadmium (Cd) is classified as a heavy metal, and it is completely unnecessary for humans from the physiological point of view. It occurs naturally in the environment. Its presence is due to, among other things, volcanic activity, rock erosion processes and forest fires [90]. However, cadmium appears much more frequently in the environment as a result of agricultural and industrial pollution [91]. Owing to the fact that it is highly toxic for humans (it causes, among other things, a number of cancers), its presence in the environment is carefully studied [92]. The presence of cadmium in the bottom sediments of water bodies exhibits considerable regional variation. Concentrations of this metal are frequently so low as to be undetectable, for instance, in the bottom sediments of the Terragido dam reservoir in Portugal [62] and of Lake Volta in Ghana [93]. The bottom sediments of the Jianhu Lake in China contain 0.29–0.42 mg/kg of cadmium on average [56]. Cadmium was found at a similar level (0.46 mg/kg) in the sediments of the Kapshagay Reservoir in Kazakhstan [57]. In the Hoedong Reservoir located in South Korea, which is used as a source of drinking water, cadmium was present in amounts ranging from 1.4 to 1.8 mg/kg [49]. In the sediments of Lake Taihu—China’s third-largest freshwater lake, which is used for fishing, recreation and as a supply of drinking water—cadmium concentrations range from 0.23 to 3.07 mg/kg [58]. In several reservoirs impounded by dams in Germany, the average cadmium content in bottom sediments amounted to 4.03 mg/kg [59]. Against the backdrop of the presented variation in cadmium concentrations, its presence in the bottom sediments of water bodies in the Silesian Upland and its periphery, which ranges from 0.7 to 286 mg/kg, is unique in the world. The extremely high concentrations of cadmium in sediments and the toxicity of this metal have already been highlighted on numerous occasions with reference to water bodies situated in the vicinity of non-ferrous smelters [12,40].

Nickel (Ni) is widely used in many industries, and thus, human and environmental exposure to nickel compounds is ubiquitous [94]. Many negative health impacts have been demonstrated in connection with human exposure to this metal. Carcinogenic and allergic effects are the most commonly observed ones [95]. On the Silesian Upland and its periphery, elevated nickel concentrations in bottom sediments are mostly associated with human activity. This element is present in the bottom sediments of water bodies used for recreation purposes in amounts ranging from 10 to 115 mg/kg. Although these levels generally exceed those considered natural, higher nickel concentrations are not uncommon in the world. In the Polish Lake Łebsko, which is the largest lake in the southern Baltic coastal zone, the range of nickel concentrations is from 13.7 to 184.4 mg/kg [63]. Much higher nickel concentrations—from 139 to 666 mg/kg, with an average for the entire water body of 305 mg/kg—are found in the bottom sediments of the Badovc dam reservoir in Kosovo, which is the source of drinking water for Pristina [64]. These relatively high values are explained by the geological structure of the area in question.

Copper (Cu) is easily dissolved and migrates in solutions; on the other hand, it is bound by organic matter and clay minerals, and is easily precipitated [96]. As a micronutrient, it is essential for the proper development and functioning of the human body. Both copper deficiency and excess have adverse health effects [97]. In the bottom sediments of the examined water bodies in the Silesian Upland, copper was found in amounts ranging from 11 to 298 mg/kg. At the lower end, the range corresponds to the geochemical background level, whereas the upper values are among the highest concentrations found in sediments worldwide. Elevated concentrations of copper in the environment are found especially in copper mining and processing areas. An example in this regard is the Ružín Reservoir in eastern Slovakia, which is mainly used for recreational purposes and also for industrial water supply. Its catchment contains old, flooded mine areas and spoil heaps left by the mining and smelting of, among others, copper ores. Copper concentrations ranging from 196.0 to 310.7 mg/kg have been found in the bottom sediments of this reservoir [65]. Typically, copper concentrations in sediments are not high.

Cobalt (Co) has a variety of (mainly industrial) applications [98]. As a basic component of vitamin B12, it is an essential micronutrient for humans [99]. In humans, cobalt deficiency is most often observed, but there are some cases of cobalt poisoning [100]. In the bottom sediments of water bodies used for recreational purposes in the Silesian Upland, cobalt is present in amounts ranging from 3 to 40 mg/kg, and its levels do not differ substantially from those found in other water bodies around the world, although its concentration in sediment traps is many times higher. For example, in sediments of tailings ponds that operated in an open-pit nickel mining area in the southeastern part of the Indonesian island of Celebes, cobalt was found at levels of 255 mg/kg [68]. Usually, however, these concentrations are significantly (by an order of magnitude) lower.

Chromium (Cr) is present in numerous minerals [101], and its compounds are used primarily in various industries, mainly chemical and metallurgical. Environmental pollution by this metal is commonly traced back to human industrial activity [102]. Chromium has long been recognized as a toxic, mutagenic and carcinogenic metal [103]. Chromium concentrations in bottom sediments of water bodies exhibit considerable variation both within individual water bodies and among different regions of the world. In the bottom sediments of water bodies in the Silesian Upland, chromium was found in amounts ranging from 22 to 203 mg/kg, with a median of 101.5 mg/kg. Similar variation can be observed in other water bodies around the world. Extremely high chromium concentrations of 882.2 mg/kg in bottom sediments were found in an artificial water body (pond) used for the farming of fish (tilapia) in the eastern part of Kolkata in India. In these areas, it is common practice to use municipal wastewater to feed such ponds. This is particularly important in the context of harvesting fish for consumption, as it may have an effect on human health [75].

Arsenic (As) occurs naturally in nature: it is present, inter alia, in volcanic ash and rocks, forming a variety of minerals [104]. It is commonly accumulated in coal, lignite and oil deposits [105], and also accompanies sulfur [106] and metal deposits [107]. Anthropogenic arsenic pollution is mainly due to coal combustion and metal smelting [108,109]. Arsenic is also used in fertilizers, herbicides and insecticides [110]. Human exposure to arsenic occurs mainly through the consumption of water [111] and seafood, especially shellfish [112]. Arsenic can cause serious skin diseases, including skin cancer; lung, bladder and kidney cancer; cardiovascular diseases; hypertension; and diabetes [6,111]. It also appears to have an adverse impact on reproduction—e.g., by causing infant mortality [112]. Arsenic content in the bottom sediments of water bodies worldwide averages 5 mg/kg [113], but it varies regionally. Thus, these arsenic concentrations are in line with its levels in the bottom sediments of water bodies used for recreational purposes in the Silesian Upland and its periphery, but only with respect to the minimum (8 mg/kg), mean (36.5 mg/kg) and median (20 mg/kg) values. With maximum arsenic concentration of 178 mg/kg, the water bodies studied occupy a unique position in the world.

Barium (Ba) is a metal that naturally occurs in the environment in very low concentrations. This element and its compounds are mainly used in industry and agriculture [114]. Reported health effects of exposure to barium include cardiovascular and kidney diseases; and metabolic, neurological and psychiatric disorders [115]. Barium was found in the bottom sediments of water bodies used for recreational purposes in the Silesian Upland and its periphery in amounts ranging from 263 to 19,300 mg/kg, the median of which was 459 mg/kg. This median level is several times higher than the regional geochemical background but is in line with natural concentrations in sediments worldwide. Apart from the very high concentration of barium in the sediments of one of the water bodies (19,100–19,300 mg/kg), its concentrations in the sediments of the other water bodies correspond to those found in other parts of the world.

Antimony (Sb) and its compounds occur naturally in the Earth’s crust, from which they are released into the environment. Antimony has found uses mainly in industry. Its toxicity to humans most often manifests itself in the form of respiratory irritation, antimoniosis (a form of pneumoconiosis), skin spots and gastrointestinal problems [116]. If only for these reasons, the Sb concentrations found in the bottom sediments of the water bodies studied, ranging from 0.9 to 52.5 mg/kg, should be considered hazardous, especially in the context of the fact that its natural content in rocks ranges from a few tenths of a milligram per kilogram to one point five milligrams per kilogram [41,44,45,46]. Although Sb is among those trace elements whose levels in bottom sediments of water bodies are rarely studied, there are reports of much higher antimony concentrations in such sediments. For example, the metal was present at 258.8–466.6 mg/kg in the sediments of the Lengshuigou dam reservoir in the upper reaches of the Duliu River in southwestern China [82]. The average antimony content in the bottom sediments of the Goczałkowice Reservoir on the Vistula River in southern Poland, which is the primary source of drinking water for a polycentric agglomeration of several million people, ranged from 80 to 120 mg/kg [83].

Under natural conditions, bromine (Br) occurs in a liquid state. Its geochemical circulation is closely related to the circulation of water in nature. The highest concentrations of bromine are found in salt deposits [96]. Bromine has found a use in flame retardant chemicals, which are widely used in producing electronics, plastics and textiles. Bromine toxicity typically manifests itself in the form of diabetes, developmental disorders, cancer and changes in thyroid function [117]. For these reasons, it should be borne in mind that the amounts of bromine found in the bottom sediments studied, ranging from 1 to 31 mg/kg, are at least several times higher than the levels considered natural.

Strontium (Sr) is a naturally occurring alkaline earth metal exhibiting high mobility and reactivity. Its presence in the environment is also associated with human activities, e.g., nuclear fallout, mineral fertilizers and industrial activity [118]. Excessive strontium intake can cause abnormal skeletal development, bone calcification and increased bone fragility [119]. In the bottom sediments of the studied limnic sites in the Silesian Upland, strontium was recorded at a wide range of levels from 63 to 510 mg/kg, but the natural level in the region is 24 mg/kg [42]. Nationwide, it is 20 mg/kg [43], and it is 300–375 mg/kg worldwide [41,44,45,46]. For instance, concentrations of 94–99 mg/kg are found in sediments in water bodies in Argentina [79]. Slightly higher strontium levels, between 186 and 274 mg/kg, have been detected in the Irkutsk Reservoir [81]. Significantly higher concentrations of this metal (averaging 706 mg/kg) were identified in the sediments of the Kouris Reservoir in Cyprus. The lake’s waters are used for drinking water, irrigation and recreation [84].

Sulphur (S) occurs on Earth in its native form, and it also forms various chemical compounds. As an important component of many biomolecules, it is essential for the proper functioning of the human body. Its toxicity is mainly related to high sulfur dioxide levels, which adversely affect human health, causing bronchitis and respiratory problems [120]. In the studied bottom sediments of water bodies used for recreational purposes in the Silesian Upland and its periphery, sulfur occurs in concentrations ranging from 0.001 to 4.590%, with a median of 0.692%, i.e., typically much higher than the geochemical background values.

### 5.2. Interpretation of Geochemical Indicators

The bottom sediments of water bodies used for recreational purposes in the Silesian Upland vary in terms of the levels of metals, non-metals and metalloids both within individual water bodies and between them (Table 2 and Table 3). Differences in trace element concentrations often reach several orders of magnitude, as reflected by the following concentrations: Pb (30–3020 mg/kg), Zn (142–35,300 mg/kg), Cd (0.7–286 mg/kg), Ni (10–115 mg/kg), Cu (11–298 mg/kg), Co (3–40 mg/kg), Cr (22–203 mg/kg), As (8–178 mg/kg), Ba (263–19,300 mg/kg), Sb (0.9–52.5 mg/kg), Br (1–31 mg/kg), Sr (63–510 mg/kg) and S (0.001–4.590%). This is due not so much to natural factors (since, for instance, substrate formations of lake basins are lithologically homogeneous), but primarily to anthropogenic ones, such as industrial processes taking place in catchment areas and transport side effects. [1]. Metals, non-metals and metalloids are present in the bottom sediments of water bodies used for recreational purposes in the Silesian Upland and its periphery in amounts that usually exceed concentrations found in other water bodies, and for some elements, their levels are record highs, unprecedented among bodies of water in the world (e.g., cadmium—286 mg/kg, zinc—35,300 mg/kg, lead—3020 mg/kg and arsenic—178 mg/kg) (Table 6).

Geoaccumulation index values in bottom sediments reflect the wide variation in the presence of the metals, metalloids and non-metals analyzed (Table 4). The entire qualitative spectrum of bottom sediments is present, from the absence of contamination to extreme contamination levels, as reflected by the range of values found: −6.31 < *I_geo_* < 10.90. For such elements as Sr, Co, Cr, Ni and Cu, the bottom sediments can be described as free of contamination, or, with some exceptions, slightly contaminated. However, in stark contrast is the heavy or even extreme contamination of bottom sediments with cadmium, and in the case of some reservoirs, also with Pb, Zn, As, Sb and S. Intermediate sediment contamination levels were found with respect to Br. In terms of geoaccumulation index values, the extreme contamination of bottom sediments of three water bodies intensively used for recreational purposes, i.e., the Stawiki, Morawa and Gliniak, stands out. This is a consequence of the long-standing operation of a non-ferrous metal smelter in the vicinity of the water bodies and the storage of metallurgical waste or its use for various purposes in the catchment area of the water bodies, and even within their basins. In the catchment area of these water bodies, 51% is categorized as industrial and urban area (Figure 3). The concentrations of trace elements contrast sharply with their significantly lower concentrations in other water bodies with lithologically similar basin substrates (e.g., Pławniowice and Rogoźnik). In general, for any water body, there is a greater or lesser relationship between the level of bottom-sediment contamination and the use of the catchment area, but there are also many apparent relationships. The Chechło reservoir, located amidst forests and with a catchment area devoid of industrial areas, is within a short distance from a zinc smelter, and bottom-sediment contamination is likely to be caused mainly by atmospheric deposition. The acidification of the environment in the vicinity of the reservoir affects the mobility of metals and the reduced potential for their accumulation in sediments [121], despite its location in the vicinity of a significant non-ferrous metallurgical industry zone. The remaining water bodies are also under varying degrees of anthropogenic stress. Many water bodies show intermediate levels of contamination by non-metals and metalloids—in relation to the described extreme cases—whose concentrations in bottom sediments exceed the geochemical background values. The data also prove that the cascading locations of water bodies along watercourses affect the concentrations of metals, non-metals and metalloids in bottom sediments. Although it is not a 100% dependable correlation, the sediments of the first basin of the cascading stream development tend to be most polluted with trace elements, and the last basin had lower concentrations—see the Pogoria and Rogoźnik reservoir complexes. This demonstrates the significant variation in the factors determining the occurrence of the elements analyzed (Figure 3).

The values of the contamination factor ($C_{f}^{i}$) and contamination degree (*C_d_*) put forward by L. Håkanson [38] confirm the poor qualitative status of the sediments. The former index varied for individual trace elements, ranging from no contamination or low sediment contamination ($C_{f}^{i}$ < 1), through moderate contamination (1 ≤ $C_{f}^{i}$ < 3), to significant contamination (3 ≤ $C_{f}^{i}$ < 6) and very heavy contamination ($C_{f}^{i}$ > 6). The minimum values of this indicator for each of the substances studied indicated no contamination or low contamination, but the maximum values were (with the exception of Cr and PCBs) indicative of contamination, i.e., 0.2–6.0 (Cu), 0.4–43.1 (Pb), 0.8–201.7 (Zn), 0.7–286.0 (Cd), 0.2–2.3 (Cr), 0.6–13.7 (As), 0.0–1.1 (PCBs) and 0.0–3.0 (Hg). On the other hand, the sediment contamination degree (*C_d_*) was in the range 4.6 < *C_d_* < 513.1, and only for one reservoir, used for recreational purposes, was it determined as low (*C_d_* < 8). For four water bodies, it was significant (16 ≤ *C_d_* < 32), and for another five it was very high (*C_d_* > 32); for the five remaining water bodies, it varied, oscillating between the minimum and maximum values (Figure 2). The accumulation of toxic metals, non-metals and metalloids in the bottom sediments of the studied water bodies is an environmental problem of natural and social significance. This is in line with A. T. Jankowski et al. [32] reporting the elimination of eel populations in the Morawa reservoir and the high mortality of tench in a neighboring reservoir. In both cases, heavy metal contamination is considered the likely cause. This is in line with the opinion of M. Kostecki [122], who stated that heavy metal contamination of some ecosystems of water bodies in the Silesian Upland already poses a threat to human health; and in the phyto- and zooplankton, vascular plant vegetation and ichthyofauna, the recorded concentrations should be categorized as contamination-level.

The ratios of the values measured to the regional geochemical background values make it possible to quantify the different concentrations of elements in the bottom sediments of the water bodies studied, taking into account the levels considered natural in the region of the Silesian Upland and its periphery, and at the same time indirectly indicating the levels of contamination of these sediments. The *I_RE_* index also varied significantly for individual elements, and its ranges were as follows: Cu: 0.7–19.9, Pb: 0.5–51.2, Zn: 0.5–136.3, Ni: 0.9–10.5, Cd: 0.3–114.4, Co: 0.8–10.0, Cr: 2.4–22.6, Ba: 2.7–196.9, Sr: 2.6–21.3, As: 1.3–29.7 and S: 0.0–88.3. These ratios of the concentrations found to the regional geochemical background indicate sediment contamination for virtually each element listed and in each of the water bodies studied (Table 5). In the case of several water bodies, very high I_RE_ values were found, suggesting the need to exclude such reservoirs from recreational use or at least to prevent any activities based on direct contact of human bodies with bottom sediments, for instance, by prohibiting the consumption of caught fish, diving and bathing. A suggested threshold for the purposes of scientific discussions concerning the suitability of a body of water for recreational use is a ratio of the levels found to the regional sediment geochemical background of not higher than *I_RE_* ≤ 5.0. At the same time, the very high values of the rate of exceeding the regional geochemical background for the measured elements in the bottom sediments provide significant grounds for engaging in reclamation activities. They should be directed at the removal of bottom sediments containing toxic metals, non-metals and metalloids. The target effect of reclamation measures should be the elimination of the threat to the environment and human health and life associated with the risk of exposure to toxic metals, non-metals and metalloids.

## 6. Conclusions

The research conducted supports several conclusions about the presence of metals and non-metals in the bottom sediments of water bodies used for recreational purposes.

The bottom sediments of water bodies used for recreational purposes in the Silesian Upland and its periphery are contaminated to varying degrees with toxic metals, metalloids and non-metals, as reflected by the values of geoecological indicators—i.e., −6.31 < *I_geo_* < 10.90, 0.0 ≤ $C_{f}^{i}$ < 286.0, 4.6 ≤ *C_d_* < 513.1 and 0.5 < *I_RE_* < 196.9. Sediment contamination is a consequence of human activity, and for Cd, Zn, Pb and As, the values we found remain the highest in the world.

The presence of metals, non-metals and metalloids in bottom sediments should be taken into account when classifying water bodies as suitable for recreational use, independently of the hydrochemical indicators used to date when assessing such suitability. In particular, the presence of certain toxic metals, non-metals and metalloids (e.g., lead, zinc, cadmium, chromium, strontium and arsenic) in bottom sediments should be monitored, and the threshold for water body suitability for recreational purposes should be a ratio of the levels found to the geochemical background of *I_RE_* ≤ 5.0.

The water bodies used for recreational purposes in the Silesian Upland and its periphery do not meet the geoecological conditions for their safe use in terms of recreation and leisure activities. Due to the fact that regional geochemical background levels of metals, non-metals and metalloids in their bottom sediments were exceeded multiple times over, forms of their recreational use that directly affect the participants’ health (e.g., fishing and the consumption of fish and other aquatic organisms) should be abandoned. In order to eliminate the threat to the environment and human health, it is necessary to undertake reclamation measures involving the removal of bottom sediments.

## Figures and Tables

**Table 1:** Morpho- and hydrometric parameters of the water bodies in the Silesian Upland.

Water Body Name	Geographical Coordinates		TC	MA	EC	WR	NO_3_^−^	PO_4_^3−^	Recreational Functions of the Water Body
	Latitude	Longitude	[dam^3^]	[ha]	[μS/cm]	[pH]	[mg/dm^3^]		
Dzierżno Małe	50°23′16.30″ N	18°33′51.10″ E	12,600	160.0	679.0	7.5–8.7	37.1	0.09	S, C, F, W, M, B, O
Pogoria I	50°21′27.00″ N	19°14′15.00″ E	3600	75.0	736.0	7.8–8.5	36.6	0.04	S, C, F, M, B, N, O
Pogoria III	50°21′13.11″ N	19°12′05.00″ E	12,000	208.0	483.0	7.4–8.2	38.3	0.04	S, C, F, D, M, B, O
Chechło	50°28′04.00″ N	18°54′49.10″ E	1300	90.0	183.0	6.9–7.6	8.4	0.02	S, C, F, M, B, O
Stawiki	50°16′25.56″ N	19°06′35.59″ E	131	7.6	784.5	7.9–8.3	22.0	0.11	C, F, W, M, B, N, O
Morawa	50°16′24.56″ N	19°07′19.57″ E	693	34.7	380.0	8.0–8.6	35.5	2.82	S, C, F, M, B, N, O
Gliniak	50°15′53.55″ N	19°07′00.54″ E	824	38.7	512.1	8.0–8.4	6.3	0.04	S, C, F, W, M, B, N, O
Sosina	50°14′27.00″ N	19°19′50.05″ E	1000	50.0	547.4	8.0–8.6	31.3	1.32	S, C, F, W, M, B, O
Pławniowice	50°23′29.23″ N	18°28′08.00″ E	29,100	240.0	617.0	7.8–9.1	9.8	0.08	S, C, F, W, M, B, O
Mały Zalew	50°23′20.45″ N	18°29′55.77″ E	143	6.5	488.0	7.6–8.2	18.9	3.43	C, F, W, M, B
Rogoźnik II	50°24′13.40″ N	19°02′40.03″ E	340	25.0	651.0	8.0–8.2	24.3	0.09	F, M, B, O
Rogoźnik I	50°23′54.59″ N	19°01′43.58″ E	360	12.1	644.0	7.9–8.5	14.0	0.11	S, C, F, M, B, O
Balaton	50°16′31.21″ N	19°15′11.16″ E	71	9.0	535.5	7.9–8.2	25.1	0.11	C, F, M, B, O
Koparki	50°13′42.52″ N	19°18′40.77″ E	440	4.0	707.0	8.1–8.7	4.5	0.00	D, B, O
Paprocany	50°05′05.59″ N	18°59′02.22″ E	1600	110.0	315.0	7.3–8.4	14.2	0.14	S, C, F, W, M, B, N, O

Explanations: TC—total capacity, MA—maximum area, EC—electrolytic conductivity, WR—water reaction, S—sailing, C—canoeing, F—fishing, D—diving, W—water sports, M—swimming, B—beach and waterfront recreation, N—nature conservation within the water body, O—others.

**Table 2:** Basic statistical characteristics of metal and non-metal concentrations in the bottom sediments of water bodies used for recreational purposes in the Silesian Upland and its periphery.

Parameter	Cu	Pb	Zn	Ni	Cd	Co	Cr	Ba	Sr	As	Sb	Br	S
	[mg/kg]												[%]
Minimum	11.0	30.0	142.0	10.0	0.7	3.0	22.0	261.0	63.0	8.0	0.9	1.0	0.001
25% quartile	19.5	56.5	302.3	19.5	2.1	10.5	59.8	407.5	112.5	13.0	1.4	4.8	0.038
Median	25.0	145.0	940.0	34.0	11.9	17.0	101.5	459.0	149.0	20.0	1.9	10.0	0.692
75% quartile	55.5	425.5	1390.0	54.0	17.6	25.5	122.0	654.5	236.5	41.0	5.5	20.0	1.670
Maximum	298.0	3020.0	35,300.0	115.0	286.0	40.0	203.0	19,300.0	510.0	178.0	52.5	31.0	4.590
Arithmetic mean	56.4	481.8	3204.7	39.2	27.7	19.0	94.4	1795.2	191.3	36.5	6.7	12.4	1.043
Standard deviation	71.4	813.9	7316.7	23.0	54.6	10.2	42.8	4824.8	116.2	39.0	11.2	9.2	1.134

**Table 3:** Concentrations of metals, non-metals and metalloids in the bottom sediments of water bodies used for recreational purposes in the Silesian Upland and its periphery.

No. of Water Bodies (see Figure 1)	Parameter	Cu	Pb	Zn	Ni	Cd	Co	Cr	Ba	Sr	As	Sb	Br	S
		[mg/kg]												[%]
1	min	19	56	288	19	2.1	10	45	386	203	12	1.5	13.0	0.001
	max	28	88	480	32	3.3	17	86	483	434	13	2.0	27.0	1.170
2	min	26	46	232	44	1.2	21	122	563	89	9	1.2	1.0	0.001
	max	67	429	2338	54	29.0	23	150	750	114	30	5.6	10.0	0.001
3	min	12	35	142	19	0.7	11	92	388	100	8	0.9	1.0	0.001
	max	58	467	1220	61	13.1	26	118	668	127	36	7.7	28.0	0.347
4	min	63	478	1360	40	16.0	14	78	19,100	476	39	5.4	11.0	0.500
	max	79	510	1480	56	20.0	34	105	19,300	510	44	8.8	14.0	0.790
5	min	123	1070	5940	47	56.9	33	120	459	152	45	19.6	10.0	2.040
	max	156	2250	9210	56	99.9	35	203	480	235	92	37.8	24.0	3.330
6	min	204	2580	13,800	96	119.0	34	153	741	151	105	31.6	7.0	2.750
	max	298	3020	35,300	115	119.0	40	182	817	209	135	52.5	10.0	4.590
7	min	250	2560	16,300	67	172.0	23	130	524	155	152	20.4	10.0	3.240
	max	270	2680	28,900	73	286.0	38	140	659	165	178	28.3	17.0	3.510
8	min	47	265	1630	62	17.5	28	109	394	194	20	3.3	6.0	1.690
	max	48	293	1690	67	17.6	33	113	409	206	23	3.5	6.0	1.890
9	min	18	30	165	18	1.8	8	36	347	227	14	1.4	18.0	1.320
	max	21	36	199	18	1.9	10	62	385	286	20	1.4	30.0	2.100
10	min	25	51	1025	36	13.0	18	57	430	131	32	1.6	23.0	0.610
	max	35	63	1035	38	15.7	20	65	474	149	32	2.1	31.0	0.880
11	min	20	408	762	20	16.9	10	67	619	119	30	2.6	3.4	0.900
	max	24	422	838	22	25.7	14	77	678	132	34	2.6	4.6	1.000
12	min	11	225	418	10	11.4	5	50	411	140	11	1.4	5.0	0.500
	max	17	237	450	14	12.3	7	60	421	142	17	1.4	5.0	0.660
13	min	22	134	1001	30	11.9	15	122	409	83	13	1.6	3.0	0.692
	max	22	145	1033	34	12.5	15	127	449	96	14	1.7	3.0	0.730
14	min	16	162	1090	12	5.5	3	22	261	63	50	1.4	9.0	0.074
	max	19	166	1130	13	6.9	3	23	262	65	63	1.5	13.0	0.080
15	min	18	49	693	34	5.4	25	122	502	102	23	1.8	4.0	0.640
	max	35	96	799	51	6.4	40	157	694	136	48	3.1	6.0	1.990

**Table 4:** Ranges of geoaccumulation index values for metals and non-metals in the bottom sediments of water bodies used for recreational purposes in the Silesian Upland and its periphery.

No. of Water Bodies (see Figure 1)	Parameter	Cu	Pb	Zn	Ni	Cd	Co	Cr	Ba	Sr	As	Sb	Br	S
		*I_geo_*												
1	min	−1.62	1.13	1.52	−2.12	3.81	−1.35	−1.20	−1.15	−1.37	2.32	2.32	2.05	−6.31
	max	−1.06	1.79	2.26	−1.37	4.46	−0.58	−0.27	−0.82	−0.27	2.44	2.74	3.10	3.88
2	min	−1.17	0.85	1.21	−0.91	3.00	−0.28	0.24	−0.60	−2.56	1.91	2.00	−1.66	−6.31
	max	0.20	4.07	4.54	−0.61	7.59	−0.15	0.54	−0.19	−2.20	3.64	4.22	1.67	−6.31
3	min	−2.29	0.46	0.50	−2.12	2.22	−1.21	−0.17	−1.14	−2.39	1.74	1.58	−1.66	−6.31
	max	−0.01	4.19	3.60	−0.44	6.45	0.03	0.19	−0.36	−2.05	3.91	4.68	3.15	2.13
4	min	0.11	4.23	3.76	−1.04	6.74	−0.87	−0.41	4.48	−0.14	4.02	4.17	1.80	2.65
	max	0.43	4.32	3.88	−0.56	7.06	0.42	0.02	4.50	−0.04	4.20	4.87	2.15	3.31
5	min	1.07	5.39	5.89	−0.81	8.57	0.37	0.21	−0.90	−1.79	4.23	6.03	1.67	4.68
	max	1.42	6.46	6.52	−0.56	9.38	0.46	0.97	−0.83	−1.16	5.26	6.98	2.93	5.39
6	min	1.80	6.66	7.10	0.22	9.63	0.42	0.56	−0.21	−1.80	5.45	6.72	1.15	5.11
	max	2.35	6.89	8.46	0.48	9.63	0.65	0.81	−0.07	−1.33	5.81	7.45	1.67	5.85
7	min	2.10	6.65	7.34	−0.30	10.16	−0.15	0.33	−0.71	−1.76	5.98	6.09	1.67	5.35
	max	2.21	6.72	8.17	−0.18	10.90	0.58	0.44	−0.38	−1.67	6.21	6.56	2.43	5.46
8	min	−0.32	3.38	4.02	−0.41	6.87	0.13	0.07	−1.12	−1.44	3.06	3.46	0.93	4.41
	max	−0.29	3.52	4.07	−0.30	6.87	0.37	0.13	−1.06	−1.35	3.26	3.54	0.93	4.57
9	min	−1.70	0.23	0.72	−2.20	3.58	−1.67	−1.52	−1.30	−1.21	2.54	2.22	2.51	4.05
	max	−1.48	0.48	0.99	−2.20	3.66	−1.35	−0.74	−1.15	−0.88	3.06	2.22	3.25	4.72
10	min	−1.23	1.00	3.35	−1.20	6.44	−0.50	−0.86	−0.99	−2.00	3.74	2.42	2.87	2.94
	max	−0.74	1.30	3.36	−1.12	6.71	−0.35	−0.67	−0.85	−1.82	3.74	2.81	3.30	3.47
11	min	−1.55	4.00	2.92	−2.04	6.82	−1.35	−0.63	−0.47	−2.14	3.64	3.12	0.11	3.50
	max	−1.29	4.05	3.06	−1.91	7.42	−0.87	−0.43	−0.33	−1.99	3.82	3.12	0.55	3.65
12	min	−2.41	3.14	2.06	−3.04	6.25	−2.35	−1.05	−1.06	−1.91	2.20	2.22	0.67	2.65
	max	−1.78	3.22	2.16	−2.56	6.36	−1.87	−0.79	−1.02	−1.89	2.82	2.22	0.67	3.05
13	min	−1.41	2.39	3.32	−1.46	6.31	−0.77	0.24	−1.06	−2.66	2.44	2.42	−0.07	3.12
	max	−1.41	2.51	3.36	−1.28	6.38	−0.77	0.30	−0.93	−2.45	2.54	2.50	−0.07	3.20
14	min	−1.87	2.67	3.44	−2.78	5.20	−3.09	−2.23	−1.71	−3.06	4.38	2.22	1.51	−0.10
	max	−1.62	2.70	3.49	−2.67	5.52	−3.09	−2.17	−1.71	−3.01	4.71	2.32	2.05	0.01
15	min	−1.70	0.94	2.79	−1.28	5.17	−0.03	0.24	−0.77	−2.36	3.26	2.58	0.34	3.01
	max	−0.74	1.91	2.99	−0.69	5.42	0.65	0.60	−0.30	−1.95	4.32	3.37	0.93	4.65
Explanations:														
practically uncontaminated (class 0: *I_geo_* ≤ 0.0)														
uncontaminated to moderately contaminated (class I: 0.0 < *I_geo_* ≤ 1.0)														
moderately contaminated (class II: 1.0 < *I_geo_* ≤ 2.0)														
moderately to heavily contaminated (class III: 2.0 < *I_geo_* ≤ 3.0)														
heavily contaminated (class IV: 3.0 < *I_geo_* ≤ 4.0)														
heavily to extremely contaminated (class V: 4.0 < *I_geo_* ≤ 5.0)														
extremely contaminated (class VI: I_geo_ > 5.0)														

**Table 5:** Ranges of the values found as multiples of the regional geochemical background for metals and non-metals in the bottom sediments of water bodies used for recreational purposes in the Silesian Upland and its periphery.

No. of Water Bodies (see Figure 1)	Parameter	Cu	Pb	Zn	Ni	Cd	Co	Cr	Ba	Sr	As	Sb	Br	S
		*I_RE_*												
1	min	1.3	0.9	1.1	1.7	0.8	2.5	5.0	3.9	8.5	2.0	(–)	(–)	0.0
	max	1.9	1.5	1.9	2.9	1.3	4.3	9.6	4.9	18.1	2.2	(–)	(–)	22.5
2	min	1.7	0.8	0.9	4.0	0.5	5.3	13.6	5.7	3.7	1.5	(–)	(–)	0.0
	max	4.5	7.3	9.0	4.9	11.6	5.8	16.7	7.7	4.8	5.0	(–)	(–)	0.0
3	min	0.8	0.6	0.5	1.7	0.3	2.8	10.2	4.0	4.2	1.3	(–)	(–)	0.0
	max	3.9	7.9	4.7	5.5	5.2	6.5	13.1	6.8	5.3	6.0	(–)	(–)	6.7
4	min	4.2	8.1	5.3	3.6	6.4	3.5	8.7	194.9	19.8	6.5	(–)	(–)	9.6
	max	5.3	8.6	5.7	5.1	8.0	8.5	11.7	196.9	21.3	7.3	(–)	(–)	15.2
5	min	8.2	18.1	22.9	4.3	22.8	8.3	13.3	4.7	6.3	7.5	(–)	(–)	39.2
	max	10.4	38.1	35.6	5.1	40.0	8.8	22.6	4.9	9.8	15.3	(–)	(–)	64.0
6	min	13.6	43.7	53.3	8.7	47.6	8.5	17.0	7.6	6.3	17.5	(–)	(–)	52.9
	max	19.9	51.2	136.3	10.5	47.6	10.0	20.2	8.3	8.7	22.5	(–)	(–)	88.3
7	min	16.7	43.4	62.9	6.1	68.8	5.8	14.4	5.3	6.5	25.3	(–)	(–)	62.3
	max	18.0	45.4	111.6	6.6	114.4	9.5	15.6	6.7	6.9	29.7	(–)	(–)	67.5
8	min	3.1	4.5	6.3	5.6	7.0	7.0	12.1	4.0	8.1	3.3	(–)	(–)	32.5
	max	3.2	5.0	6.5	6.1	7.0	8.3	12.6	4.2	8.6	3.8	(–)	(–)	36.3
9	min	1.2	0.5	0.6	1.6	0.7	2.0	4.0	3.5	9.5	2.3	(–)	(–)	25.4
	max	1.4	0.6	0.8	1.6	0.8	2.5	6.9	3.9	11.9	3.3	(–)	(–)	40.4
10	min	1.7	0.9	4.0	3.3	5.2	4.5	6.3	4.4	5.5	5.3	(–)	(–)	11.7
	max	2.3	1.1	4.0	3.5	6.3	5.0	7.2	4.8	6.2	5.3	(–)	(–)	16.9
11	min	1.3	6.9	2.9	1.8	6.8	2.5	7.4	6.3	5.0	5.0	(–)	(–)	17.3
	max	1.6	7.2	3.2	2.0	10.3	3.5	8.6	6.9	5.5	5.7	(–)	(–)	19.2
12	min	0.7	3.8	1.6	0.9	4.6	1.3	5.6	4.2	5.8	1.8	(–)	(–)	9.6
	max	1.1	4.0	1.7	1.3	4.9	1.8	6.7	4.3	5.9	2.8	(–)	(–)	12.7
13	min	1.5	2.3	3.9	2.7	4.8	3.8	13.6	4.2	3.5	2.2	(–)	(–)	13.3
	max	1.5	2.5	4.0	3.1	5.0	3.8	14.1	4.6	4.0	2.3	(–)	(–)	14.0
14	min	1.1	2.7	4.2	1.1	2.2	0.8	2.4	2.7	2.6	8.3	(–)	(–)	1.4
	max	1.3	2.8	4.4	1.2	2.8	0.8	2.6	2.7	2.7	10.5	(–)	(–)	1.5
15	min	1.2	0.8	2.7	3.1	2.2	6.3	13.6	5.1	4.3	3.8	(–)	(–)	12.3
	max	2.3	1.6	3.1	4.6	2.6	10.0	17.4	7.1	5.7	8.0	(–)	(–)	38.3
Explanations: (–)—lack of data.														
0.0 < *I_RE_* ≤ 1.0														
1.0 < *I_RE_* ≤ 5.0														
5.0 < *I_RE_* ≤ 10.0														
10.0 < *I_RE_* ≤ 100.0														
*I_RE_* > 100.0														

**Table 6:** Concentrations of metals, non-metals and metalloids in the bottom sediments of selected water bodies used for recreational purposes worldwide.

Item	Water Bodies Used for Recreational Purposes on the Silesian Upland	Water Bodies Used for Recreational Purposes Worldwide and the Concentration of Metals, Non-Metals and Metalloids
Pb	30.0–3020.0 mg/kg	Hoedong Reservoir (South Korea)—53.6–69.2 mg/kg [49]; Římov Reservoir (Czech Republic)—up to 42.0 mg/kg [50]; Lake Eğirdir (Turkey)—0.8–22.1 mg/kg [51]; the water bodies forming the Dnieper reservoir cascade (Ukraine)—from a minimum of 17.2 mg/kg (Kremenchug Reservoir) to a maximum of 63.3 mg/kg (Kakhovka Reservoir) [52].
Zn	142.0–35,300.0 mg/kg	Lake Gusinoe (Russia)—74.2–598.0 mg/kg [53]; Lake Yangzong (China)—149.2 mg/kg (average) [54]; Lake Qaroun (Egypt)—0.01–92.6 mg/kg [55].
Cd	0.7–286.0 mg/kg	Lake Jianhu (China)—0.29–0.42 mg/kg [56]; Kapshagay Reservoir (Kazakhstan)—0.46 mg/kg [57]; Hoedong Reservoir (South Korea)—1.4–1.8 mg/kg [48]; Lake Taihu (China)—0.23–3.07 mg/kg [58]; several reservoirs in Germany—4.03 mg/kg (average) [59].
Ni	10.0–115.0 mg/kg	Lake Balaton (Hungary)—4.4–5.5 mg/kg [60]; Wivenhoe Reservoir (Australia)—23.5–26.5 mg/kg; Little Nerang Reservoir (Australia)—18.5–19.0 mg/kg [61]; Terragido Reservoir (Portugal)—18.0–80.0 mg/kg [62]; Lake Łebsko (Poland)—13.7–184.4 mg/kg [63]; Badovci Lake (Kosovo)—139.0–666.0 mg/kg [64].
Cu	11.0–298.0 mg/kg	Ružín Reservoir (Slovakia)—196.0–310.7 mg/kg [65]; Kapshagay Reservoir (Kazakhstan)—0.12–0.38 mg/kg [57]; the ponds used for intensive shrimp farming (Brazil)—10.0–20.0 mg/kg [66]; Dobczyce Reservoir (Poland)—5.5–45.4 mg/kg [67].
Co	3.0–40.0 mg/kg	Tailings ponds (Indonesia)—255.0 mg/kg [68]; Kallar Kahar Lake (Pakistan)—4.03–11.34 mg/kg [69]; Wivenhoe Reservoir (Australia)—20.7–21.8 mg/kg; Little Nerang (Australia)—16.6–19.1 mg/kg [61]; Guaíba Lake (Brasil)—6.4–97.6 mg/kg [70].
Cr	22.0–203.0 mg/kg	Três Marias Reservoir (Brasil)—2.0–150.6 mg/kg [71]; Dianchi Lake (China)—68.6–95.3 mg/kg [72]; Orlík Reservoir (Czech Republic)—72.4–123 mg/kg (108 mg/kg—average) [73]; Mujib Reservoir (Jordan)—79.8–136.8 mg/kg (114.2 mg/kg—average) [74]; ponds in Kolkata (India)—up to 882.2 mg/kg [75].
As	8.0–178.0 mg/kg	Yangebup Lake (Australia)—21.8 mg/kg (average) [76]; Badovci Lake (Kosovo)—10.0–29.9 mg/kg (24.2 mg/kg—average) [64]; Rożnów Lake (Poland)—5.2 mg/kg (average) [77]; 15 lakes located on the Crimean Peninsula—from 3.05 mg/kg (Dzharylgach Lake) to 20.41 mg/kg (Adjigol Lake) [78].
Ba	263.0–19,300.0 mg/kg	Los Molinos and San Roque reservoirs (Argentina)—383–400 mg/kg [79]; Kaw Reservoir (USA)—280–420 mg/kg [80]; Irkutsk Reservoir (Russia)—582–633 mg/kg [81]; Guaíba Lake (Brasil)—139–1448 mg/kg [70].
Sb	0.9–52.5 mg/kg	Lengshuigou Reservoir (China)—258.8–466.6 mg/kg [82]; Goczałkowice Reservoir (Poland)—80.0–120.0 mg/kg [83].
Br	1.0–31.0 mg/kg	lack of data
Sr	63.0–510.0 mg/kg	Los Molinos and San Roque reservoirs (Argentina)—94.0–99.0 mg/kg [79]; Irkutsk Reservoir (Russia)—186.0–274.0 mg/kg [81]; Kouris Reservoir (Cyprus)—706.0 mg/kg (average) [84].
S	0.001–4.590%	lack of data

## Data Availability

Data sharing is not applicable to this article.
